# Assessment of temperature on mortality burden and economic impacts in a basin-located mega-city

**DOI:** 10.1371/journal.pone.0355835

**Published:** 2026-08-12

**Authors:** Dan Kuang, Wen Qian, Jingwen Sun, Jingqiu Yao, Xufang Gao, Nan Du, Jiaqi Huang, Wei Huang, Cheng Wang, Rong Lu

**Affiliations:** Department of Environmental and School Health, Chengdu Center for Disease Control and Prevention, Chengdu, Sichuan, China; University of South Carolina, UNITED STATES OF AMERICA

## Abstract

**Objective:**

This study aimed to examine the association between ambient temperature and non-accidental mortality, and to systematically quantify the mortality burden and economic impacts attributable to non-optimum temperatures in Chengdu in 2022.

**Methods:**

We collected daily non-accidental mortality records for 23 counties from the Chengdu Center for Disease Control and Prevention from January 1, 2015, to December 31, 2021, along with potential effect modifiers covering demographic, air quality, and socioeconomic indicators. A Poisson-based distributed lag non-linear model (DLNM) was used to estimate the temperature–non-accidental mortality association, followed by quantification of non-optimal temperature-related mortality risk and burden. We further evaluated the excess deaths and associated economic burden of non-optimal temperatures in Chengdu in 2022 using the value of statistical life (VSL) method.

**Results:**

During the study period, 584,063 non-accidental deaths were recorded, and a stable U-shaped temperature–mortality association was identified. In total, non-optimal temperatures accounted for 7.24% of non-accidental mortality, with a higher AF for cold temperatures (5.82%) than for hot temperatures (1.42%). The AFs for cardiovascular and respiratory disease mortality were 10.59% and 7.63%, respectively. More significant mortality burdens related to non-optimal temperatures were found in females, the elderly (≥75 years), and groups with low education or alternative marital status. In 2022, non-optimal temperature exposure led to 11,316 excess deaths in Chengdu, translating into a VSL-based mortality economic loss of 21.428 billion RMB.

**Conclusions:**

Given ongoing global climate change, the mortality burden and economic losses caused by non-optimal temperatures in this basin-located mega-city merit urgent attention. Corresponding adaptive strategies are therefore required to protect the general public, especially vulnerable populations, from worsening climate-related health risks.

## Introduction

Under the context of global climate change, extreme weather events including heatwaves and cold spells are projected to increase in frequency, intensity, and geographic coverage in the future [1–3]. Exposure to non-optimal temperatures poses substantial health risks and disease burdens globally, emerging as a critical public health threat comparable to ambient air pollution [4–6]. Accumulating epidemiological evidence has well documented that non-optimal temperature exposure adversely affects human health and elevates the risks of all-cause non-accidental mortality [7], cardiovascular disease mortality [8], and respiratory disease mortality [9].

To date, a large body of epidemiological research has explored the temporal and spatial associations between temperature exposure and mortality [8,10–12]. However, existing regional and national studies often neglect heterogeneous exposure-response relationships and temperature-attributable disease burdens across different regions, thereby restricting the generalizability of their conclusions. Located in the Sichuan Basin, Chengdu possesses unique topographic and meteorological characteristics, including terrain-induced local atmospheric circulation, humid air accumulation, frequent winter temperature inversions, and stable atmospheric conditions, which differentiate its climatic features from other regions across China [13]. Prior studies have preliminarily evaluated the economic burden attributable to extreme temperatures; for instance, the unprecedented heatwaves in China in 2017 led to 16,299 excess deaths and a mortality-related economic loss of 61.304 billion RMB [14]. Nevertheless, evidence regarding the mortality economic burden associated with non-optimal temperatures in Chengdu remains scarce. Such localized evidence is essential for optimizing public risk communication strategies and evaluating the cost-effectiveness of targeted public health interventions.

This study aimed to investigate the association between ambient temperature and non-accidental mortality, as well as to systematically quantify the mortality burden attributable to cold and hot non-optimal temperatures across diverse population subgroups. We further estimated the excess deaths and corresponding economic burden attributable to non-optimal temperatures in Chengdu in 2022. To the best of our knowledge, this is the first study to evaluate the excess mortality and economic impacts caused by non-optimal temperatures in Chengdu.

## Materials and methods

### Data collection

This study covered all 23 counties of Chengdu, covering a total resident population of 21.26 million, to explore the relationship between non-accidental mortality and non-optimal temperatures. Daily mortality records for Chengdu residents from January 1, 2015, to December 31, 2021, were retrieved from the standardized Death Surveillance System administered by the Chengdu Center for Disease Control and Prevention (CDC). All death causes were categorized according to the 10th Revision of the International Classification of Diseases (ICD-10), including total non-accidental causes (A00–R99), cardiovascular diseases (I00–I99), and respiratory diseases (J00–J98). Daily non-accidental deaths (NAD) were further stratified by multiple demographic variables: sex, age groups (0–64 years, 65–74 years, and ≥75 years), educational attainment (low: ≤ 9 years of education; high: > 9 years of education), and marital status (married or alternative marriage statuses).

Daily meteorological variables, including daily mean temperature, relative humidity, and atmospheric pressure, were collected from the Chengdu Meteorological Bureau. Daily air quality indicators, including the average concentration of fine particulate matter (PM2.5) and the daily maximum 8-hour average concentration of ozone (O3), were obtained from the Chengdu Environmental Monitoring Center. County-level data on resident population, gross domestic product (GDP), and per capita disposable income were extracted from the Chengdu Statistical Yearbook.

### Associations between temperature and mortality

Consistent with previous studies [10,15], we examined the associations between temperature and mortality by using the distribution lag non-linear model (DLNM) with a quasi-Poisson regression. The model formula was as follows:


$$\begin{array}{c} \text{Log}\left[\text{E}(\text{Yt})\right]=\alpha +\text{cb}(\text{Temperature},\text{lag})+\text{ns}(\text{relative humidity},\;\text{3})+\text{ns}(\text{Time},\text{ df}\times \text{year})+\text{DOWt} \end{array}$$ (1)

Briefly, E(Yt) denotes estimated daily NAD on day t; α is the model intercept; cb(Temperature, lag) represents the cross-base matrix of daily temperature built by DLNM, and lag refers to the maximum lag up to 14 days; ns(relative humidity, 3) is the natural cubic B spline of the present day relative humidity with three degrees of freedom. ns(Time, df*year) is the natural cubic B spline of calendar day with 7 degrees of freedom (df) per year. DOWt is an indicator variable for the day of the week on day t. A maximum lag of 14 days was selected based on biological plausibility. Given that cold-induced physiological responses may accumulate over several days and eventually trigger acute adverse health events, this lag setting is consistent with those adopted in major multi-city epidemiological studies across China. The degrees of freedom (df) for the temporal trend (7 df per year) and relative humidity (3 df) were determined via Akaike Information Criterion (AIC) optimization to balance model goodness-of-fit and overfitting risk. The temperature-mortality association was visualized as lag-cumulative relative risk (RR) exposure-response curves. Stratified analyses were further performed to explore temperature-related non-accidental mortality risks across subgroups defined by sex, age, marital status, and educational attainment. All core analyses were adjusted for potential confounding factors, including daily PM_2.5_ concentrations and daily maximum 8-hour O_3_ concentrations. Additionally, sensitivity analyses were conducted by applying alternative degrees of freedom for spline functions and alternative maximum lag periods of 7 and 21 days.

### Estimation of attributable fractions

According to the previously described method, we calculated the number of deaths on each day of the series due to temperature, using as reference and cut-off the Minimum Mortality Temperature (MMT), which was derived from the prediction of the overall cumulative exposure-response association and referred as the optimum temperature. We obtained the total counts of deaths attributed to non-optimum temperatures by summing the contributions from all the days in the series and gained the total attributable fraction by dividing the total number of deaths by the total number of attributable deaths. We also derived the attributable fractions associated with cold and heat by summing the subsets of days with temperature lower and higher than the MMT. We further calculated the attributable factions associated with extreme cold, moderate cold, moderate heat, and extreme heat according to specific percentiles of temperature distribution, which is < 2.5^th^ percentile, 2.5^th^ percentile up to the MMT, MMT to the 97.5^th^ percentile, and >97.5^th^ percentile, respectively. Finally, we obtained the confidence intervals through Monte Carlo simulations by 10000 random samples with the assumed normal distribution of the estimation coefficients [16]. It is important to note that these attributable fractions are model-based estimates derived from the statistical association between temperature and mortality, as quantified by the DLNM. They represent the theoretical reduction in mortality that would be expected if all non‑optimal temperatures were shifted to the minimum mortality temperature, assuming that the observed exposure‑response relationship is causal and that all other factors remain constant. These estimates should be interpreted as statistical projections rather than directly observable counts.

### Excess non-accidental deaths and economic impact attributable to 2022 non-optimal temperatures

We estimated the excess non-accidental mortality attributed to 2022 non-optimal temperatures for Chengdu, based on the RR estimated from the overall cumulative exposure-response association [17]. The formula was as follows to calculate the excess deaths:


$$\begin{array}{c} \text{EDi}=\text{POPi}\times \text{N}\times \frac{RR-\text{1}}{RR} \end{array}$$ (2)

Where, EDi is the estimated number of excess non-accidental deaths during 2022 in county i; POPi is the number of population in county i in 2022; N is the average daily non-accidental mortality counts in county i in 2022; RR is the relative risk of non-optimal temperature for non-accidental mortality, as estimated from the Eq. (1).

We further assessed the death-related economic impacts of the 2022 non-optimal temperature for 23 counties in Chengdu based on the estimated excess non-accidental deaths. The economic impact of 2022 non-optimal temperature was estimated by multiplying the value of statistical life (VSL) with excess deaths for each county. The VSL does not represent the monetary value of an individual life, but rather reflects a population‑based measure of how much individuals in a given population are collectively willing to pay for small reductions in mortality risk. In this study, VSL is used as a standard metric to quantify the aggregate economic burden of premature deaths attributable to non‑optimal temperatures. We used an estimated of VSL from a Chinese study in 74 cities, which adjustments for different locations using the per capita annual income of each city relative to the national average per capita annual income and an income elasticity [18]. We adjusted the baseline VSL to each county in Chengdu using per capita annual disposable income, based on the rationale that individuals’ willingness to pay for mortality risk reduction is positively correlated with their income level. This income adjustment is standard practice in VSL transfer studies and ensures that the economic burden estimates reflect the local economic context and willingness- to -pay capacity of each county’s population. The calculation equation was shown in Eq. (3).


$$\begin{array}{c} \text{VSLi}=\text{EDi}\times \left(\frac{VSL\times \text{INCOME2022i}}{INCOME2016}\right)\text{e} \end{array}$$ (3)

Where, VSLi is the estimated VSL attributable to the 2022 non-optimal temperature for city i (100 million yuan); EDi is the estimated number of excess non-accidental deaths during 2022 in county i; VSL is 1.525 million yuan adopted from the Chinese study. INCOME2022i is the per capita annual income for county i in 2022 (ten thousand yuan); INCOME2016 is 4.35 ten thousand yuan adopted from the Chinese study as the national average per capita annual income in 2016. e is 1 as the income elasticity. We further calculated the ratio of VSL to GDP in 2022 for each county. Statistical analysis was performed using R software version 4.3.2.

## Results

### Descriptive analysis

Descriptive statistics for meteorological conditions, air quality, NAD counts, and individual demographic characteristics in Chengdu from January 1, 2015, to December 31, 2021, are presented in Table 1. A total of 584,063 NAD cases were recorded during the study period, corresponding to a daily average of 228.4 deaths. Of all NAD cases, 58.6% occurred in males and 78.9% in older adults. Specifically, cardiovascular and respiratory diseases contributed to 185,981 and 141,311 NAD cases, respectively. The daily mean temperature was 16.9°C (range: −1.6°C to 30.5°C), and the daily mean relative humidity was 79.0% (range: 36.0% to 99.0%). The average concentrations of PM_2.5_ and O_3_ were 52.0 µg/m^3^ (range: 4.0–254.3 µg/m^3^) and 85.4 µg/m^3^ (range: 5.0–305.0 µg/m^3^), respectively. Notably, no missing values were observed for daily mortality, air quality, or meteorological data throughout the study period.

**Table 1:** Summary descriptive statistics on weather conditions and average number of daily NAD in Chengdu, 2015-2021.

Variables	Mean±SD	Min	P25	P50	P75	Max
Meteorological Factors						
Mean temperature (°C)	16.9 ± 7.3	−1.6	10.1	17.4	23.4	30.5
Relative humidity (%)	79.0 ± 9.6	36	73	79.7	86	99
Pressure (hpa)	949.3 ± 7.5	931.2	942.7	949.3	955.3	975.1
Wind speed (m/s)	1.4 ± 0.4	0.5	1.1	1.3	1.6	3.9
Air pollution						
PM2.5 (ug/m3)	52.0 ± 36.6	4.0	26.0	42.2	67.1	254.3
O3 (ug/m3)	92.9 ± 52.7	5.0	52.5	82.3	128.0	305.0
Non-accidental Death	228.4 ± 41.4	137	198	222	252	386
Cardiovascular	72.7 ± 17.7	36	59	70	84	140
Respiratory	55.3 ± 18.4	23	42	51	64	140
Sex						
Male	133.8 ± 24.6	77	116	130	148	232
Female	94.6 ± 19.6	49	81	91	106	173
Age						
0–64 years old	48.2 ± 8.5	21	42	48	54	82
65–74 years old	50.6 ± 10.2	23	43	50	57	88
≥ 75 years old	129.7 ± 30.3	69	107	124	147	238
Marital status						
Married	142.9 ± 23.7	86	126	140	156	236
Others	85.5 ± 20.6	39	71	82	98	169
Education						
Low	199.1 ± 37.7	119	171	193	220	346
High	29.4 ± 6.6	8	25	29	34	59

### Associations between temperature and mortality

**Fig 1:**
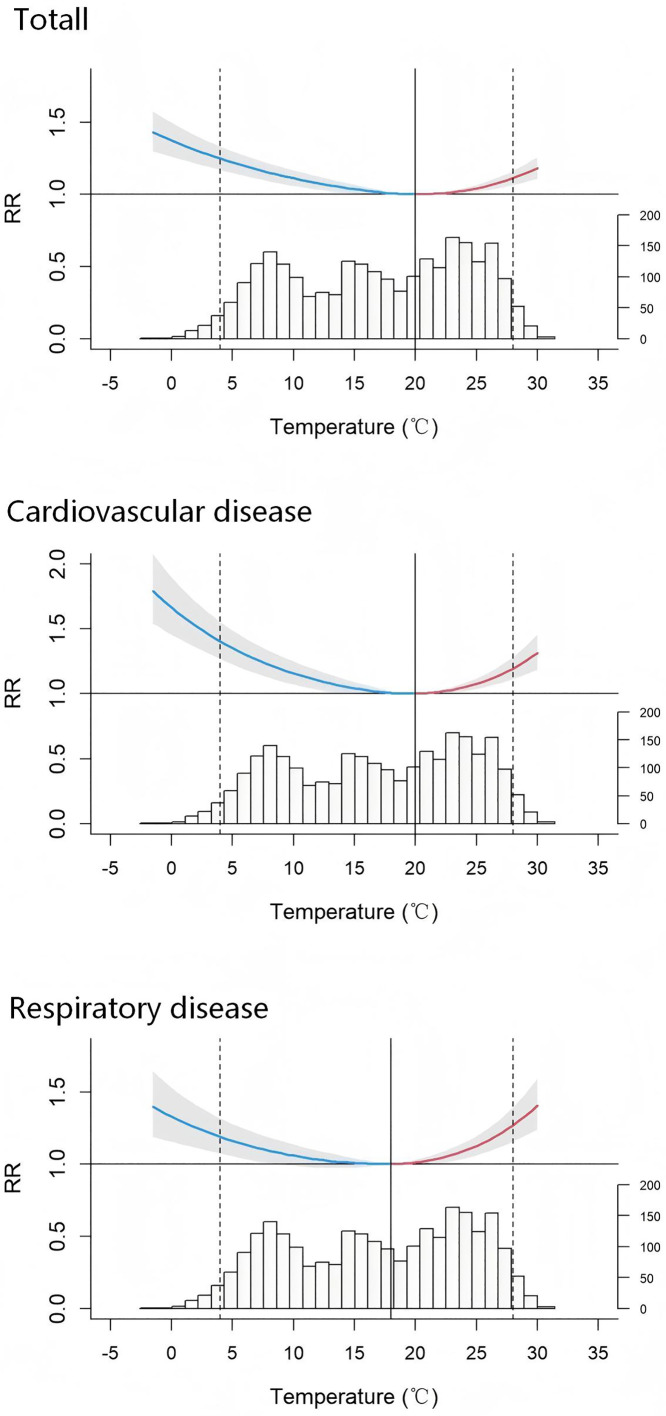
Cumulative exposure-response curves for associations between daily mean temperature and mortality over lag days 0-14 in Chengdu, 2015-2021. The blue line indicates the exposure-response association of cold (with 95% empirical confidence interval, shaded gray), and the red line presents the heat. The gray solid line is minimum mortality temperature and the dashed lines are the 2.5^th^ and 97.5^th^ percentile.

**Fig 2:**
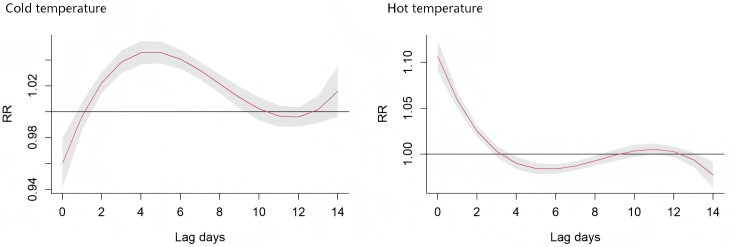
Lag structure in effects of extreme temperatures on daily mortality in Chengdu, 2015-2021. Solid line indicates the mean estimate and shaded area presents the 95% confidence intervals.

The overall temperature distribution and cumulative exposure-response associations between daily temperature and cause-specific mortality are illustrated in Fig 1. All curves presented a consistent U-shaped pattern, demonstrating elevated mortality risks under non-optimal temperature conditions. The minimum mortality temperatures were comparable for cardiovascular and respiratory disease mortality, at 20°C and 18°C, respectively. As depicted in Fig 2, the mortality risks attributable to extreme cold temperatures (4°C) generally emerged on lag day 1, peaked at lag day 4, and gradually declined until lag day 12, with mild residual effects observed in the subsequent lag days. In comparison, the excess mortality risks linked to extreme hot temperatures (28°C) were strongest on the current day, dropped sharply after lag day 2, and were followed by significant mortality displacement at lag day 6 for both cardiovascular and respiratory disease mortality outcomes.

Table 2 presents the cumulative temperature-mortality associations for different disease causes and population subgroups across the 0–14 lag days. At the city-wide level, the minimum mortality temperature was 20°C, corresponding to the 60th percentile of the local temperature distribution. The relative risk of cardiovascular mortality associated with extreme cold temperatures was higher than that for respiratory mortality. In cold temperature scenarios, males, older adults, and individuals with low educational attainment or alternative marital status exhibited greater mortality relative risks than other population subgroups. Similarly, females, older adults, and groups with low educational attainment or alternative marital status experienced higher hot temperature-related mortality risks compared with their counterparts.

**Table 2:** Relative risks of daily mortality associated with non-optimum temperatures in Chengdu, 2015-2022.

Variable	MMP (%)	MMT (°C)	RR (95%CI)	
			Extreme low	Extreme high
NAD	60	20	1.25 (1.17–1.33)	1.11 (1.06–1.16)
Cardiovascular	60	20	1.40 (1.27–1.55)	1.20 (1.11–1.28)
Respiratory	53	18	1.19 (1.08–1.32)	1.27 (1.16–1.39)
Sex				
Male	64	21	1.28 (1.18–1.39)	1.05 (1.01–1.10)
Female	56	19	1.21 (1.11–1.32)	1.22 (1.14–1.30)
Age				
0–64 years old	79	24	1.08 (0.94–1.24)	1.00 (0.95–1.06)
65–74 years old	74	23	1.15 (1.02–1.31)	1.02 (0.96–1.08)
≥ 75 years old	56	19	1.36 (1.26–1.47)	1.21 (1.14–1.28)
Marital status				
Married	69	22	1.23 (1.14–1.34)	1.03 (0.98–1.07)
Others	56	19	1.28 (1.17–1.41)	1.29 (1.20–1.39)
Education				
Low	60	20	1.26 (1.17–1.35)	1.13 (1.08–1.19)
High	85	25	1.23 (1.04–1.45)	1.01 (0.96–1.07)

Note: MMP = Minimum mortality centile of temperature distributions. MMT = Minimum mortality temperature. Extreme low = 2.5th centile of temperature distribution (4°C). Extreme high = 97.5th centile of temperature distribution (28°C).

### Attributable fractions

Table 3 summarizes the attributable fractions (AFs) of mortality for different disease outcomes and population subgroups linked to cold and hot components of non-optimal temperatures. The overall AF of non-optimal temperatures for total non-accidental mortality was 7.24% (95% CI: 5.21%–9.27%). Stratified analyses showed that cold temperatures contributed a substantially higher AF (5.82%, 95% CI: 3.48%–8.05%) than hot temperatures (1.42%, 95% CI: 0.74%–2.10%), accounting for approximately 80% of the total temperature-attributable mortality burden. In subgroup analyses, higher cold-related AFs were observed for cardiovascular disease mortality, older adults, and individuals with high educational attainment or alternative marital status. By contrast, elevated hot-related AFs were detected for respiratory disease mortality, older adults, and populations with low educational attainment or alternative marital status.

**Table 3:** The attributable fraction of mortality associated with cold and heat in Chengdu, 2015-2021.

Variables	MMP	MMT	Total (%)	Cold (%)	Heat (%)
NAD	60	20	7.24 (5.21–9.27)	5.82 (3.48–8.05)	1.42 (0.74–2.10)
Cardiovascular	60	20	10.59 (7.28–13.64)	8.24 (4.56–11.59)	2.35 (1.26–3.39)
Respiratory	53	18	7.63 (4.32–10.45)	4.25 (0.79–7.71)	3.38 (1.65–4.95)
Sex					
Male	64	21	6.94 (4.36–9.42)	6.38 (3.57–9.20)	0.57 (−0.18–1.31)
Female	56	19	7.97 (5.19–10.36)	5.27 (2.32–7.87)	2.71 (1.52–3.90)
Age					
0–64 years old	79	24	2.42 (–3.82–7.33)	2.38 (–3.78–8.04)	0.04 (−0.54–0.55)
65–74 years old	74	23	5.40 (0.17–9.76)	5.20 (0.10–9.84)	0.21 (−0.51–0.88)
≥ 75 years old	56	19	10.29 (8.11–12.36)	7.77 (5.15–10.12)	2.53 (1.51–3.49)
Marital status					
Married	69	22	5.79 (2.95–8.75)	5.53 (2.41–8.40)	0.27 (−0.35–0.84)
Others	56	19	10.33 (7.56–12.89)	6.86 (3.97–9.74)	3.48 (2.30–4.61)
Education					
Low	60	20	7.47 (5.15–9.58)	5.76 (3.26–7.95)	1.72 (0.96–2.43)
High	85	25	7.82 (1.17–13.98)	7.74 (1.12–13.69)	0.09 (−0.34–0.54)

Note: MMP = Minimum mortality centile of temperature distributions. MMT = Minimum mortality temperature.

Sensitivity analysis results are illustrated in S2 Table. Overall, the AFs of non-optimal temperatures for non-accidental mortality remained stable across alternative lag structures (7 and 21 days) and in models with or without adjustment for PM_2.5_ and O_3_. The total AFs ranged from 6.18% to 7.24% across all modeling scenarios, and cold-attributable mortality fractions consistently exceeded heat-attributable fractions in all sensitivity specifications. These findings demonstrate the robustness of our primary results to variations in model settings and confounding adjustment strategies.

**Fig 3:**
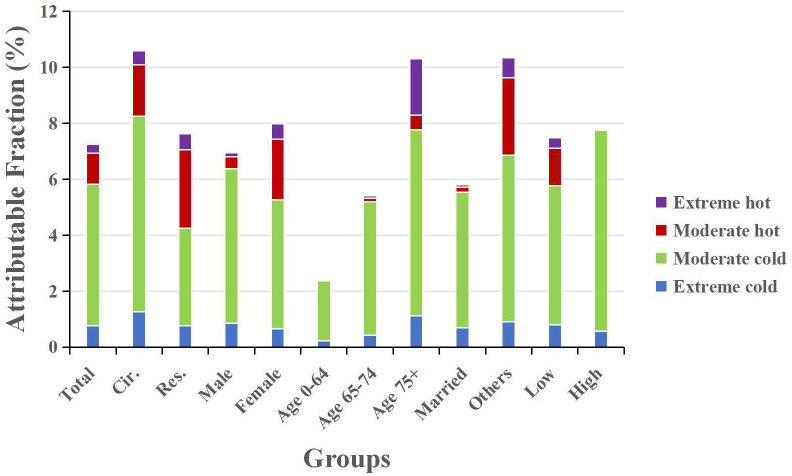
Attribution fractions of mortality due to moderate and extreme non-optimum temperatures. Moderate cold temperatures range from 4 to 20°C, moderate heat temperatures range from 20 to 28°C, extreme cold temperatures range from −1.6 to 4°C, and extreme heat temperatures range from 28 to 30.5°C.

We further decomposed the total non-optimal temperature-attributable mortality burden into four temperature categories: moderate cold (4°C-20°C), moderate heat (20°C-28°C), extreme cold (−1.6°C-4°C), and extreme heat (28°C-30.5 °C). Stratified category analysis indicated that moderate cold contributed the largest proportion of attributable mortality fractions (45.7%−88.7%). In comparison, extreme cold and extreme heat only accounted for a minor share of mortality burden, with subgroup-specific AFs ranging from 7.2%−12.2% and 0.6%−19.5%, respectively (Fig 3).

### Excess death and economic impact of 2022

**Fig 4:**
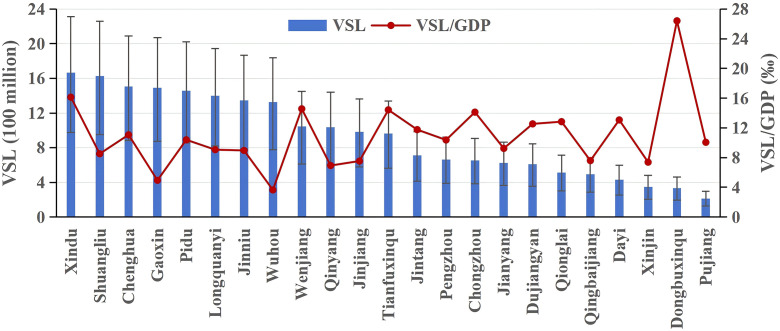
The economic burden attributed to the 2022 non-optimum temperatures in Chengdu. Economic burden was quantified as the value of statistical life (VSL), as shown in the right. Blue bars show VSL, and the error bars show the 95% confidence intervals for VSL. Red line show VSL as of GDP (%).

The following estimates of excess deaths and economic burden were model‑based projections derived from the exposure‑response association estimated during the 2015–2021 period, applied to the 2022 population and temperature distribution. They represented the expected number of deaths attributable to non‑optimal temperatures under the assumption that the historical exposure‑response relationship remains applicable to the 2022 population. Using the mortality data, the estimated relative risk of mortality associated with non-optimal temperatures corresponds to a total of 11,316 excess deaths in 2022. The mortality burden was notably severe in counties located in the second ring region and central Chengdu, accounting for 69.8% (7,897/11,316) of the excess deaths. Among the 23 counties, Xindu had the highest estimated number of excess deaths, with 839 deaths in 2022 attributable to non-optimal temperatures. The death burden attributable to non-optimal temperatures in 2022 was noticeably lower in the third ring region (Pujiang, Xinjin, and Dongbuxinqu) compared to other regions (S3 Table). Fig 4 illustrates the economic impacts related to deaths attributable to non-optimal temperatures in 2022. The total VSL loss from non-optimal temperatures in 2022 was 21.428 billion RMB. Xindu, Shuangliu, Chenghua, Gaoxin, and Pidu ranked in the top five counties with the highest economic burden related to non-optimal temperatures in 2022. The average ratio of VSL relative to GDP was 8.70‰, ranging from 3.65‰ in Wuhou (excess deaths: 649; VSL: 1,324 million RMB) to 26.40‰ in Dongbuxinqu (excess deaths: 213; VSL: 334 million RMB).

## Discussion

This study evaluated the exposure-response relationships between ambient temperature and mortality from total non-accidental causes, cardiovascular diseases, and respiratory diseases in Chengdu. We further quantified the mortality burden attributable to cold and hot non-optimal temperatures across diverse population subgroups. Additionally, we estimated the excess deaths and corresponding economic losses linked to non-optimal temperatures for the year 2022. Our core findings revealed that 7.24% of all non-accidental mortality could be attributed to non-optimal temperatures, with cold temperatures responsible for approximately 80% of this total disease burden.

Notably, these attributable burden estimates represent statistical projections generated from the observed exposure–response relationships, and thus rely on the core assumptions embedded within the distributed DLNM framework. Stratified subgroup analyses identified females, adults aged ≥75 years, and people with low educational attainment or alternative marital status as the most vulnerable populations. In addition, an estimated 11,316 excess deaths in Chengdu in 2022 were attributable to non-optimal temperatures, corresponding to an overall mortality-related economic loss of 21.428 billion RMB.

The present findings should be interpreted in the context of Chengdu’s unique geographical and climatic characteristics. As a city situated in the topographically enclosed western Sichuan Basin and surrounded by the Qionglai and Longquan mountain ranges, Chengdu features distinct local meteorological conditions that differ from those of other Chinese cities; the surrounding mountain barriers restrict atmospheric circulation, triggering frequent autumn and winter temperature inversions and the accumulation of air pollutants and atmospheric moisture. The study-period annual average relative humidity of 79.0% reflects the basin’s humid climate, which aggravates physiological stress induced by non-optimal temperatures under both cold and hot conditions: high humidity increases air thermal conductivity to accelerate bodily heat loss and amplify cold stress in cool environments, while it inhibits sweat evaporation and cutaneous heat dissipation, elevating the risk of heat-related illnesses in hot environments. Additionally, the bowl-shaped basin terrain strengthens the urban heat island effect by limiting horizontal air movement and trapping surface heat, especially during summer nights, further exacerbating the health hazards of hot temperatures for vulnerable populations in densely urbanized areas. These unique geographical and climatic interactions jointly explain the prominent mortality burden attributable to non-optimal temperatures observed in this study, highlighting the need for targeted, context-specific public health adaptation strategies that fully account for the combined effects of temperature, humidity, and air pollution.

Our results identified a U-shaped temperature-mortality association, where both cold and hot conditions elevated non-accidental mortality risks. Cold-related adverse health effects exhibited a longer lag duration than heat-related effects, which aligns with most previous single-city and multi-city epidemiological investigations [19–21]. We further observed that extreme cold temperatures yielded higher relative risks of cardiovascular mortality compared with extreme hot temperatures, consistent with existing evidence [8,22]. From a biological perspective, cold exposure triggers vasoconstriction, elevated blood pressure, increased blood viscosity, and systemic inflammatory reactions, collectively precipitating acute cardiovascular events [23]. Notably, extreme hot temperatures exerted more pronounced adverse impacts on respiratory disease mortality in our analysis, whereas several prior studies have reported contradictory outcomes [24,25]. Such heat-driven respiratory risks may be jointly attributed to elevated ambient ozone concentrations, indoor mold and dust mite exposure, and heightened atmospheric humidity [26–28]. Specifically, co-exposure to heatwaves and ozone substantially amplifies respiratory mortality risk; frequent summer heavy rainfall and flooding further raise ambient humidity levels, worsening indoor allergen exposure and deteriorating respiratory function.

This study further quantified the mortality burden attributable to non-optimal temperatures. The overall attributable fraction of total non-accidental mortality linked to non-optimal temperatures reached 7.24%, a figure comparable to the 9.21% documented in southwest China’s Guiyang [19], yet lower than the 14.33% from a national nationwide analysis [8] and the 10.86% reported in another Sichuan Basin-based study [20]. Such discrepancies can be explained by divergences in study periods, model parameter settings, as well as regional disparities in demography, climate and socioeconomic conditions. Consistent with prior literature [8,13,20], our findings identified cold temperatures, particularly moderate cold, as the primary contributor to temperature-related mortality burden. This pattern can be attributed to a higher share of cold days, a right-shifted MMT at the 60th temperature percentile (20°C), and the lagged, cumulative adverse health impacts of cold exposure, which exert stronger and longer-lasting effects than heat exposure [8]. The prominent cold-related mortality risks may stem from drastic diurnal and indoor-outdoor temperature swings that undermine cardiovascular stability [29], alongside higher transmission rates of respiratory pathogens including influenza, Mycoplasma pneumoniae and pertussis during cold seasons [30]. A prior local study covering 2011–2014 in Chengdu reported cold-attributable mortality of 9.96% versus heat-attributable mortality of merely 0.97%, marking a nearly tenfold gap [13]. By comparison, our analysis spanning 2015–2021 observed a drop in cold-related AF to 5.82%, accompanied by a rise in heat-related fraction to 1.42%. Against the backdrop of global warming, summer heatwaves have grown more frequent and intense nationwide. In 2022, China’s annual average temperature was 0.62°C higher than the 1991–2020 climate normal, with record-breaking temperatures recorded across spring, summer and autumn [31]. Meanwhile, rapid urban expansion has intensified the urban heat island effect across China [32], highlighting the urgent practical value of strengthening public adaptive capacity to extreme heat.

We further conducted stratified analyses to compare temperature-attributable mortality fractions across subgroups defined by sex, age, marital status and educational attainment. Cold-related AFs were higher among males, while females bore greater heat-attributable mortality burdens, a sex disparity consistent with existing epidemiological evidence [33]. Such gender gaps can be explained by divergent physiological characteristics, lifestyles and daily behaviors. For instance, females exhibit higher core temperature thresholds and distinct sweating patterns that limit evaporative heat dissipation under hot weather [34,35]; by contrast, males are more likely to adopt risky behaviors during cold spells, including insufficient warm clothing, low mask-wearing compliance, and delayed medical treatment upon initial symptom onset [36,37]. Older adults presented substantially higher mortality fractions attributable to non-optimal temperatures, in line with the majority of prior investigations [8,38]. This vulnerability arises from a higher prevalence of chronic comorbidities and impaired thermoregulatory function across both cold and hot environments: the elderly display weakened peripheral vasoconstriction and reduced metabolic heat generation when exposed to cold, alongside diminished sweating, cutaneous vasodilation, cardiac output adjustment and visceral blood redistribution under heat stress [39,40]. Notably, adults aged ≥75 years suffered more severe health impacts from extreme heat than extreme cold, underscoring the necessity of targeted prevention and intervention strategies for senior populations during heatwaves. Prior work has also identified marital status and education as key social determinants of temperature-related mortality risk [41,42]. Our subgroup results revealed that individuals with alternative marital status (single, separated, divorced or widowed) and low educational attainment carried higher heat-attributable mortality fractions. Alternative marital status and low education are often linked to underlying poor physical health, chronic physiological stress, substandard housing conditions and social isolation, which collectively heighten population vulnerability during heat exposure [38].

To the best of our knowledge, this study is the first to assess the economic burden associated with non-accidental mortality caused by non-optimal temperatures in Chengdu. To date, research on the economic losses related to non-optimal in China remains limited and has primarily focused on exposure to heat waves [14,43]. For example, based one study evaluating the health costs associated with non-optimal temperatures in China in 2020, the economic losses due to non-optimal temperatures accounted for 2.18% of the GDP (2,210.843 billion/ 101,598.62 billion Chinese yuan, using the VSL approach) [17]. Yan et al. reported that an overall death-related economic loss of 61.304 billion Chinese yuan was attributable to the exceptional heatwaves in China in 2017 [14]. However, it was difficult to compare our results with those of previous studies due to differences in study regions, time periods, exposure-response function coefficients, and the VSL metric used. In this study, we calculated the local exposure-response function and estimated the economic burden of non-optimal temperatures. The results showed that deaths attributable to non-optimal temperatures caused enormous economic losses (1 billion Chinese yuan) across all 23 counties in Chengdu in 2022. Moreover, the pattern of the ratio of VSL to GDP varied significantly across the 23 counties. For example, the economic impact resulted in a much smaller GDP loss in Wuhou compared to Dongbuxinqu (2.15% versus 15.60%). These findings suggest that implementing adaptation strategies tailored to local conditions, such as heatwave and cold surge warning systems, air conditioning use, and green spaces, would yield significant health and economic benefits.

To the best of our knowledge, this is the first study to quantify the economic burden of non-optimal temperature-attributable non-accidental mortality in Chengdu. To date, nationwide research estimating economic losses linked to non-optimal temperatures in China remains scarce and largely concentrates on heatwave exposure [14,43]. For instance, a national assessment of temperature-related health costs across China in 2020 estimated that economic losses induced by non-optimal temperatures made up 2.18% of national GDP (2,210.843 billion CNY out of a total GDP of 101,598.62 billion CNY) using the VSL method [17]. Yan et al. further documented that the unprecedented national heatwaves in 2017 generated total mortality-related economic losses of 61.304 billion CNY [14]. Direct comparisons between our results and prior literature are hindered by discrepancies in study coverage, research periods, exposure-response function parameters, and adopted VSL valuation standards. Unlike previous national-scale investigations, the present study constructed region-specific exposure-response curves to evaluate the economic burden driven by non-optimal temperatures. Our calculations indicated that mortality attributable to non-optimal temperatures brought substantial economic losses totaling 21.428 billion CNY across all 23 counties of Chengdu in 2022. In addition, the VSL-to-GDP ratio exhibited striking disparities among county-level administrative divisions: Wuhou District recorded a mild GDP loss ratio of 3.65%, while Dongbuxinqu reached as high as 26.40%. These results demonstrate that locally customized adaptive interventions, including early warning systems for heatwaves and cold surges, accessible cooling facilities, and urban green space construction, can deliver prominent public health gains and reduce temperature-related economic costs.

This study makes several novel contributions to the existing literature on temperature-related mortality. First, to the best of our knowledge, this is the first study to systematically quantify the economic burden of non-optimal temperature-attributable mortality in Chengdu using the VSL approach. The economic dimension, which is critical for informing cost-effectiveness evaluations of public health interventions and climate adaptation policies, has remained unexplored. Second, our county-level spatial analysis reveals significant intra-urban heterogeneity in both mortality burden and economic impact across Chengdu’s 23 counties, with the VSL-to-GDP ratio ranging from 3.65‰ in Wuhou to 26.40‰ in Dongbuxinqu-a granularity that enables locally-tailored adaptation strategies. Third, our study updates and extends the temporal evidence base with a longer and more recent time series (2015–2021). This update is particularly valuable given the accelerating pace of climate change and rapid urbanization. Collectively, these contributions extend beyond simple local estimation and provide policy-relevant evidence for climate change adaptation in rapidly urbanizing mega-cities.

This study offers several notable innovations to the existing body of evidence on temperature-related mortality. First, to the best of our knowledge, this is the first study to systematically quantify the economic burden of mortality attributable to non-optimal temperatures in Chengdu using the VSL framework. This economic perspective has long been absent from local temperature health research, yet it is essential for supporting cost-effectiveness assessments of public health interventions and optimizing climate adaptation policy formulation. Second, our fine-scale county-level spatial analysis identified substantial intra-urban heterogeneity in temperature-attributable mortality burdens and corresponding economic impacts across Chengdu’s 23 administrative counties. Such refined spatial evidence provides a solid foundation for developing differentiated, locality-targeted climate adaptation strategies. Third, this study updates and enriches the temporal evidence base by adopting a relatively recent and prolonged observational period (2015–2021), which is particularly valuable amid ongoing climate change acceleration and rapid urbanization processes. Collectively, these advances extend beyond localized epidemiological estimation and generate refined, policy-relevant evidence for climate risk adaptation in densely urbanized mega-cities.

This study has several limitations that should be acknowledged. First, temperature data were obtained from fixed outdoor monitoring stations, which may introduce exposure measurement errors compared with individual-level ambient exposure recordings. Second, this ecological study is subject to inherent methodological constraints, including the limited capacity to fully control for unmeasured confounding factors and the potential ecological fallacy. Third, only mortality outcomes were included in the current analysis, while other health endpoints such as disease morbidity and hospital visits were not incorporated. This narrow outcome coverage may underestimate the overall health and economic losses attributable to non-optimal temperatures.

## Conclusions

This study confirmed a robust U-shaped association between ambient temperature and non-accidental mortality in Chengdu. Both cold and hot non-optimal temperatures significantly elevated mortality risks, with moderate cold temperatures contributing the predominant proportion of the total temperature-related mortality burden. Subgroup analyses identified females, older adults, and individuals with alternative marital status or low educational attainment as the most vulnerable populations exposed to non-optimal temperatures. Substantial excess mortality and considerable corresponding economic losses were attributable to non-optimal temperature conditions in Chengdu in 2022. These findings highlight the urgent need to strengthen targeted public health interventions against climate-related health hazards and implement localized adaptation strategies to mitigate temperature-attributable disease burden and protect population health under ongoing climate change.

## Supporting information

S1 Table The raw data needed to replicate the findings of this study. (XLSX)

S2 Table The results of sensitivity analysis. (XLSX)

S3 Table The total excess deaths and economic burden attributed to the 2022 non-optimum temperatures in Chengdu. (XLSX)
