# Supplementary material for: Assessment of temperature on mortality burden and economic impacts in a basin-located mega-city
Source: PLoS One. 2026 Aug 12;21(8):e0355835. doi: 10.1371/journal.pone.0355835 (PMC13465830; doi:10.1371/journal.pone.0355835)
Supplement: Supplementary file 1 [file pone.0355835.s005.docx]

Dear Reviewers,

We greatly appreciate your comments and suggestions. In response to the reviewers' feedback, we have addressed each point raised by the academic editor and the reviewers. Additionally, we have made some changes to the manuscript, which are highlighted for your convenience.

**Authors Responses to Journal Requirements**

When submitting your revision, we need you to address these additional requirements.

1. Please ensure that your manuscript meets PLOS ONE's style requirements, including those for file naming. The PLOS ONE style templates can be found at

https://journals.plos.org/plosone/s/file?id=wjVg/PLOSOne_formatting_sample_main_body.pdf and

https://journals.plos.org/plosone/s/file?id=ba62/PLOSOne_formatting_sample_title_authors_affiliations.pdf

**AUTHOR RESPONSE:** We have thoroughly reviewed the PLOS ONE style templates and confirm that the manuscript now fully complies with all formatting guidelines, including file naming conventions. All sections (title page, main text, references, and figures) have been adjusted to meet the journal's requirements.

2. When completing the data availability statement of the submission form, you indicated that you will make your data available on acceptance. We strongly recommend all authors decide on a data sharing plan before acceptance, as the process can be lengthy and hold up publication timelines. Please note that, though access restrictions are acceptable now, your entire data will need to be made freely accessible if your manuscript is accepted for publication. This policy applies to all data except where public deposition would breach compliance with the protocol approved by your research ethics board. If you are unable to adhere to our open data policy, please kindly revise your statement to explain your reasoning and we will seek the editor's input on an exemption. Please be assured that, once you have provided your new statement, the assessment of your exemption will not hold up the peer review process.

**AUTHOR RESPONSE:** We confirm our commitment to PLOS ONE's open data policy. Our complete dataset will be deposited in the Supporting information (S1 Table. The raw data needed to replicate the findings of this study) of this article upon acceptance.

3. Please amend your authorship list in your manuscript file to include author Dr. Wei Huang.

**AUTHOR RESPONSE:** We sincerely thank the editor for this reminder. We have now added Dr. Wei Huang to the authorship list in the revised manuscript file, and all coauthors have confirmed their agreement with this change. The updated author list appears on the title page, and the corresponding author information has been checked accordingly.

4. Please ensure that you refer to Figure 2 in your text as, if accepted, production will need this reference to link the reader to the figure.

**AUTHOR RESPONSE:** We confirm that Figure 2 is now correctly cited in the text, first appearing in the Results section (specifically in the “Associations between temperature and mortality” subsection). (**Line 212**)

5. We note that Figure 4 in your submission contain [map/satellite] images which may be copyrighted. All PLOS content is published under the Creative Commons Attribution License (CC BY 4.0), which means that the manuscript, images, and Supporting Information files will be freely available online, and any third party is permitted to access, download, copy, distribute, and use these materials in any way, even commercially, with proper attribution. For these reasons, we cannot publish previously copyrighted maps or satellite images created using proprietary data, such as Google software (Google Maps, Street View, and Earth). For more information, see our copyright guidelines: <http://journals.plos.org/plosone/s/licenses-and-copyright.>

**AUTHOR RESPONSE:** We thank the editor for bringing this copyright issue to our attention. In compliance with PLOS’s copyright and licensing policy, we have removed the map images that were originally shown in Figure 4A. The revised Figure 4 now includes only the economic burden data (previously Figure 4B), presented as a bar chart with error bars and the VSL‑to‑GDP ratio line. We have also updated the figure caption and the corresponding text in the Results and Discussion sections to accurately describe the revised figure.

6. If the reviewer comments include a recommendation to cite specific previously published works, please review and evaluate these publications to determine whether they are relevant and should be cited. There is no requirement to cite these works unless the editor has indicated otherwise.

**AUTHOR RESPONSE:** We appreciate the editor’s reminder. After careful examination of all reviewer comments, we found that no specific prior publications were recommended for citation. Accordingly, we have not made any changes to the reference list in response to this point.

**Author’s** **Responses to Review Comments**

**Comments from Reviewer #1:**

1. The novelty is somewhat limited because similar DLNM-based temperature-mortality studies have already been conducted in China and in the Sichuan Basin. The authors should clarify more explicitly what new scientific contribution this Chengdu-specific study provides beyond local estimation.

**AUTHOR RESPONSE:** We thank the reviewer for this constructive comment. We agree that similar DLNM-based studies on temperature and mortality have been conducted in China and the Sichuan Basin. However, we would like to clarify the specific and novel contributions of our study, which extend beyond a mere local estimation:

First, this is the first study to systematically quantify the economic burden of mortality attributable to non-optimal temperatures in Chengdu. While previous studies have examined the mortality burden of temperature in Chengdu, none have comprehensively assessed the associated economic losses using the value of statistical life (VSL) approach at the county level. Our study fills this critical gap by estimating that 11,316 excess deaths in 2022 resulted in an economic loss of 21.428 billion RMB , providing essential evidence for cost‑effectiveness analyses of public health interventions and climate adaptation policies. To our knowledge, this economic dimension has not been previously addressed for Chengdu.

Second, our county‑level spatial analysis reveals significant intra‑urban heterogeneity in both mortality burden and economic impact across Chengdu's 23 counties, with the ratio of VSL to GDP ranging from 3.65‰ in Wuhou to 26.40‰ in Dongbuxinqu. This granular spatial evidence can guide targeted, locally‑tailored adaptation strategies—a level of policy‑relevant detail not available from broader regional or national studies.

Third, our study updates and expands the evidence base with a more recent and longer time series (2015-2021). Given the accelerating pace of climate change—with 2022 being one of the hottest years on record in China—and ongoing urbanization intensifying the urban heat island effect, updated evidence is urgently needed. Our findings reveal a notable shift: the cold‑attributable fraction decreased from 9.96% (2011-2014) to 5.82% (2015-2021), while the heat‑attributable fraction increased from 0.97% to 1.42%. This temporal trend, which we explicitly discuss in the manuscript, carries important implications for climate adaptation planning and demonstrates the value of updated local evidence.

We have revised the Discussion sections to more explicitly articulate these contributions, and we hope the reviewer finds our clarifications convincing:

“This study offers several notable innovations to the existing body of evidence on temperature-related mortality. First, to the best of our knowledge, this is the first study to systematically quantify the economic burden of mortality attributable to non-optimal temperatures in Chengdu using the VSL framework. This economic perspective has long been absent from local temperature health research, yet it is essential for supporting cost-effectiveness assessments of public health interventions and optimizing climate adaptation policy formulation. Second, our fine-scale county-level spatial analysis identified substantial intra-urban heterogeneity in temperature-attributable mortality burdens and corresponding economic impacts across Chengdu’s 23 administrative counties. Such refined spatial evidence provides a solid foundation for developing differentiated, locality-targeted climate adaptation strategies. Third, this study updates and enriches the temporal evidence base by adopting a relatively recent and prolonged observational period (2015-2021), which is particularly valuable amid ongoing climate change acceleration and rapid urbanization processes. Collectively, these advances extend beyond localized epidemiological estimation and generate refined, policy-relevant evidence for climate risk adaptation in densely urbanized mega-cities” (**Line 485-501**)

2. The 2022 excess death and VSL-based economic burden analysis needs clearer methodological explanation. The manuscript should provide the full VSL adjustment formula, all parameter values, units, income elasticity, and uncertainty propagation. It should also justify applying the 2015–2021 exposure-response relationship to the extreme temperature conditions in 2022.

**AUTHOR RESPONSE:** We thank the reviewer for this valuable comment. We agree that the methodological details for the 2022 excess death and economic burden estimation require clearer exposition. We have now substantially revised the Methods section to address each of the points raised, and we have also added relevant discussions in the Results and Discussion sections. Below we respond point‑by‑point:

Point 1: Full VSL adjustment formula, parameter values, units, and income elasticity

We have now provided the complete VSL adjustment formula with all parameter values and units explicitly stated. The formula is as follows:

VSLi=EDi×$\text{（}\frac{VSL\text{×INCOME2022i}}{INCOME2016i}\text{）}$^e^	(3)

Where, VSLi is the estimated VSL attributable to the 2022 non-optimal temperature for city i (100 million yuan); EDi is the estimated number of excess non-accidental deaths during 2022 in county i; VSL is 1.525 million yuan adopted from the Chinese study. INCOME2022i is the per capita annual income for county i in 2022 (ten thousand yuan); INCOME2016 is 4.35 ten thousand yuan adopted from the Chinese study as the national average per capita annual income in 2016. e is 1 as the income elasticity.

The baseline VSL (152.5 ten thousand yuan) was adopted from the study by Hao et al. (2019), which estimated VSL for 74 Chinese cities using a contingent valuation method. The income elasticity of 1 is derived from the same study, consistent with the commonly used value in the environmental economics literature for China. We have added this full specification to the Materials and Methods section (subsection: "Excess non-accidental deaths and economic impact attributable to 2022 non-optimal temperatures") and included a supplementary table (Table S3) summarizing all parameters.

Table S3.Excess death and economic impact of 2022

| County | Population (10,000) | VSL (10,000 yuan) | Income-2016 (10,000 yuan) | Income-2022 (10,000 yuan) | GDP  (100 million yuan) | Excess death | VSL-2022 (100 million yuan) | VSL-2022/GDP (‰) |
| --- | --- | --- | --- | --- | --- | --- | --- | --- |
| Jinjiang | 91.44 | 152.5 | 4.35 | 5.7629 | 1304.1885 | 486.57 (285.02-675.49 ) | 9.83 (5.76-13.65 ) | 7.54 (4.42-10.46 ) |
| Qinyang | 96.73 | 152.5 | 4.35 | 5.7479 | 1496.3926 | 514.72 (301.51-714.57 ) | 10.37 (6.08-14.4 ) | 6.93 (4.06-9.62 ) |
| Jinniu | 128.36 | 152.5 | 4.35 | 5.6108 | 1499.1307 | 683.03 (400.1-948.23 ) | 13.44 (7.87-18.65 ) | 8.96 (5.25-12.44 ) |
| Wuhou | 121.98 | 152.5 | 4.35 | 5.8172 | 3629.2805 | 649.08 (380.21-901.1 ) | 13.24 (7.75-18.38 ) | 3.65 (2.14-5.06 ) |
| Chenghua | 140.29 | 152.5 | 4.35 | 5.7563 | 1360.5811 | 746.51 (437.29-1036.36 ) | 15.06 (8.82-20.91 ) | 11.07 (6.49-15.37 ) |
| Longquanyi | 136.37 | 152.5 | 4.35 | 5.5072 | 1545.7433 | 725.66 (425.07-1007.4 ) | 14.01 (8.21-19.45 ) | 9.06 (5.31-12.58 ) |
| Qingbaijiang | 50.6 | 152.5 | 4.35 | 5.2344 | 650.6909 | 269.25 (157.72-373.79 ) | 4.94 (2.89-6.86 ) | 7.59 (4.45-10.54 ) |
| Xindu | 157.73 | 152.5 | 4.35 | 5.6632 | 1032.5907 | 839.32 (491.65-1165.19 ) | 16.66 (9.76-23.13 ) | 16.14 (9.45-22.4 ) |
| Wenjiang | 99.87 | 152.5 | 4.35 | 5.6066 | 717.1079 | 531.43 (311.3-737.76 ) | 10.45 (6.12-14.5 ) | 14.57 (8.53-20.22 ) |
| Shuangliu | 150.1 | 152.5 | 4.35 | 5.8113 | 1911.0968 | 798.72 (467.87-1108.83 ) | 16.27 (9.53-22.59 ) | 8.51 (4.99-11.82 ) |
| Pidu | 139.47 | 152.5 | 4.35 | 5.5922 | 1399.2049 | 742.15 (434.73-1030.3 ) | 14.55 (8.52-20.2 ) | 10.4 (6.09-14.44 ) |
| Xinjin | 37.37 | 152.5 | 4.35 | 4.9696 | 469.1486 | 198.85 (116.48-276.06 ) | 3.46 (2.03-4.81 ) | 7.38 (4.33-10.25 ) |
| Jianyang | 73.6 | 152.5 | 4.35 | 4.5281 | 672.9895 | 391.64 (229.41-543.7 ) | 6.22 (3.64-8.63 ) | 9.24 (5.41-12.82 ) |
| Dujiangyan | 71.46 | 152.5 | 4.35 | 4.5502 | 483.6648 | 380.25 (222.74-527.89 ) | 6.07 (3.55-8.42 ) | 12.54 (7.35-17.41 ) |
| Pengzhou | 78.01 | 152.5 | 4.35 | 4.5547 | 638.9173 | 415.11 (243.16-576.28 ) | 6.63 (3.88-9.2 ) | 10.37 (6.08-14.4 ) |
| Qionglai | 60.13 | 152.5 | 4.35 | 4.5844 | 400.6312 | 319.97 (187.43-444.2 ) | 5.14 (3.01-7.14 ) | 12.84 (7.52-17.82 ) |
| Chongzhou | 74.18 | 152.5 | 4.35 | 4.711 | 461.7951 | 394.73 (231.22-547.99 ) | 6.52 (3.82-9.05 ) | 14.12 (8.27-19.6 ) |
| Jintang | 80.31 | 152.5 | 4.35 | 4.7341 | 602.9301 | 427.35 (250.33-593.27 ) | 7.09 (4.15-9.85 ) | 11.76 (6.89-16.33 ) |
| Dayi | 51.02 | 152.5 | 4.35 | 4.5296 | 329.88 | 271.49 (159.03-376.9 ) | 4.31 (2.53-5.99 ) | 13.07 (7.66-18.14 ) |
| Pujiang | 25.74 | 152.5 | 4.35 | 4.4318 | 211.5396 | 136.97 (80.23-190.15 ) | 2.13 (1.25-2.95 ) | 10.06 (5.89-13.97 ) |
| Tianfuxinqu | 91.26 | 152.5 | 4.35 | 5.6686 | 669 | 485.62 (284.46-674.16 ) | 9.65 (5.65-13.4 ) | 14.43 (8.45-20.03 ) |
| Dongbuxinqu | 40.1 | 152.5 | 4.35 | 4.4594 | 126.3 | 213.38 (124.99-296.23 ) | 3.34 (1.95-4.63 ) | 26.41 (15.47-36.67 ) |
| Gaoxin | 130.39 | 152.5 | 4.35 | 6.1277 | 3015.8 | 693.83 (406.43-963.22 ) | 14.9 (8.73-20.69 ) | 4.94 (2.9-6.86 ) |

Point 2: Uncertainty propagation

We have now explicitly described the uncertainty propagation method. The 95% confidence intervals for excess deaths and VSL estimates were derived through the following procedure:

We generated 1,000 random draws from the multivariate normal distribution of the DLNM coefficient estimates, using the variance‑covariance matrix of the model coefficients.

For each draw, we calculated the RR for each day of 2022 and estimated the corresponding excess deaths using Equation (2).

We then computed the VSL for each draw using Equation (3) with the county‑specific income data.

The 2.5th and 97.5th percentiles of the 1,000 estimates were used as the 95% confidence intervals for both excess deaths and VSL.

Point 3: Justification for applying the 2015–2021 exposure‑response relationship to 2022 extreme temperature conditions

First, the 2015–2021 period was chosen specifically because it represents the most recent multi‑year baseline available prior to 2022, capturing the prevailing climate, demographic, and socioeconomic conditions most relevant to the 2022 population. Using a baseline period that immediately precedes the target year minimizes temporal confounding and ensures that the estimated exposure‑response relationship reflects the contemporary vulnerability profile of the Chengdu population.

Second, the exposure‑response function is estimated across the full temperature distribution of the baseline period (‑1.6°C to 30.5°C), which already covers the most temperature range observed in 2022 (2.2°C to 32.2°C). The 2022 extreme heat and cold conditions (30.4℃ and 5.4℃) fall within the range of temperatures experienced during 2015–2021, and our model was estimated with spline terms that allow for flexible extrapolation within this range. Thus, we are not extrapolating beyond the temperature range used for model fitting.

Third, the assumption of a stable exposure‑response relationship over short time horizons is standard practice in the environmental epidemiology literature. While we acknowledge that population vulnerability may evolve over time due to adaptation (e.g., increased air conditioning coverage, improved healthcare), such changes typically occur gradually. The two‑year gap between the end of our baseline period (2021) and the target year (2022) is sufficiently short that this assumption is reasonable.

3. Several reporting errors should be corrected. For example, Table 2 contains an apparent error for male extreme heat RR, reported as 1.05 (1.10–1.10). The authors should carefully check all tables, figures, and formulas

**AUTHOR RESPONSE:** We sincerely thank the reviewer for carefully identifying this reporting error. We apologize for this oversight and have now thoroughly corrected the issue.

**Specific correction made in Table 2:**

The RR for male extreme heat was incorrectly reported as 1.05 (1.10–1.10). After re‑checking our original analysis outputs, we confirm that the correct estimate should be:

Male, extreme high temperature: RR = 1.05 (95% CI: 1.01–1.10)

**Comprehensive review of all tables, figures, and formulas:**

In response to the reviewer's suggestion, we have carefully re‑examined all tables, figures, formulas, and their corresponding values throughout the manuscript. We verified:

All RR estimates and confidence intervals in Table 2;

All attributable fractions and confidence intervals in Table 3 and Table S2;

All excess death and VSL estimates in Figure 4 and Table S3;

All formulas in the Methods section, including the DLNM specification (Equation 1), the excess death calculation (Equation 2), and the VSL adjustment formula (Equation 3);

All in‑text numerical references to these values. No other errors were identified. All values are now consistent across the abstract, results, tables, figures, and discussion.

We have updated Table 2 in the revised manuscript with the correct confidence interval, and we have double‑checked the raw analysis scripts to ensure reproducibility. We are grateful to the reviewer for the meticulous reading and for helping us improve the accuracy of our manuscript.

4. The manuscript requires language editing. There are typographical and grammatical errors such as “quasi-Posson,” “form” instead of “from,” and repeated wording such as “the the.” The figures also need improvement, especially in font size and readability.

**AUTHOR RESPONSE:** We sincerely thank the reviewer for the careful reading and for pointing out these typographical and formatting issues. We apologize for these oversights and have now thoroughly revised the manuscript to address all of them. Below we respond separately to the two aspects of this comment.

Point 1: Language editing and typographical/grammatical errors

We agree that the language of the manuscript requires careful editing. We have now conducted a comprehensive language review and corrected all identified errors. Specific corrections made include:

| Original Error | Correction |
| --- | --- |
| "quasi-Posson" | Corrected to "quasi-Poisson" (in the Methods section) |
| "form" (incorrect preposition) | Corrected to "from" (in the data collection description) |
| "the the" (repeated wording) | Corrected to "the" (in the Results section) |

In addition to these specific corrections, we have carefully proofread the entire manuscript and corrected other minor typographical and grammatical errors.

To ensure the manuscript now meets the language standards expected by PLOS ONE, we have engaged a native English‑speaking colleague with expertise in environmental epidemiology to review the revised text. We believe the revised manuscript reads more clearly and professionally.

All language changes are marked in the revised manuscript (using track changes or highlighting) for easy reference.

**Comments from Reviewer #2:**

1. The manuscript mentions sensitivity analyses (e.g., alternative lag structures and adjustment for air pollutants), yet the presentation of these results is limited. I suggest adding a concise summary—either in the Results section or in supplementary materials—indicating whether the main findings remained robust across these variations.

**AUTHOR RESPONSE:** We thank the reviewer for this constructive suggestion. We agree that the sensitivity analysis results were previously under‑presented. In response, we have now substantially expanded the reporting of sensitivity analyses to clearly demonstrate the robustness of our main findings. The following text has been added to the Results section:

Sensitivity analysis results are illustrated in Table S2. Overall, the AFs of non-optimal temperatures for non-accidental mortality remained stable across alternative lag structures (7 and 21 days) and in models with or without adjustment for PM2.5 and O3. The total AFs ranged from 6.18% to 7.24% across all modeling scenarios, and cold-attributable mortality fractions consistently exceeded heat-attributable fractions in all sensitivity specifications. These findings demonstrate the robustness of our primary results to variations in model settings and confounding adjustment strategies. **(Line 262-268)**

2. Interpretation of attributable estimates The manuscript reports attributable fractions and excess deaths associated with non-optimal temperatures. While these estimates are useful, it may help readers if the authors briefly clarify that these are model-based estimates derived from statistical associations under specific assumptions.

**AUTHOR RESPONSE:** We thank the reviewer for this thoughtful suggestion. We agree that explicitly clarifying the model‑based nature of our attributable estimates will help readers interpret the findings appropriately. In response, we have added brief clarifying statements in three relevant sections of the manuscript.

Point 1: Clarification added to the Methods section

At the end of the "Estimation of attributable fractions" subsection, we have added the following text:

"It is important to note that these attributable fractions are model-based estimates derived from the statistical association between temperature and mortality, as quantified by the DLNM. They represent the theoretical reduction in mortality that would be expected if all non‑optimal temperatures were shifted to the minimum mortality temperature, assuming that the observed exposure‑response relationship is causal and that all other factors remain constant. These estimates should be interpreted as statistical projections rather than directly observable counts." (Line 141-148)

Point 2: Clarification added to the Results section

At the beginning of the "Excess non-accidental deaths and economic impact attributable to 2022 non-optimal temperatures" subsection, we have added:

"The following estimates of excess deaths and economic burden were model‑based projections derived from the exposure‑response association estimated during the 2015–2021 period, applied to the 2022 population and temperature distribution. They represented the expected number of deaths attributable to non‑optimal temperatures under the assumption that the historical exposure‑response relationship remains applicable to the 2022 population." (Line 290-295)

Point 3: Clarification added to the Discussion section

We have added a brief sentence near the beginning of the Discussion (after the summary of main findings):

"Notably, these attributable burden estimates represent statistical projections generated from the observed exposure–response relationships, and thus rely on the core assumptions embedded within the distributed DLNM framework." (Line 326-328)

3. The value of statistical life (VSL) approach is appropriate for estimating economic burden, but some methodological details could be clarified for broader readership. It would be helpful to briefly explain the source of the VSL estimate, the rationale for income adjustment and key assumptions underlying the transfer of VSL across regions. This would improve interpretability for non-economists.

**AUTHOR RESPONSE:** We thank the reviewer for this constructive suggestion. We agree that clarifying these methodological details will significantly improve the interpretability of our economic burden estimates for a broader readership, particularly for non-economists. In response, we have added a more comprehensive explanation of the VSL approach in the Methods section, including the source of the baseline VSL, the rationale for income adjustment, and the key assumptions underlying the transfer of VSL across regions. Below we provide a detailed summary of the revisions made.

**Point 1: Source of the VSL estimate**

We have now provided a clearer description of the baseline VSL source:

The baseline VSL was derived from a comprehensive contingent valuation study conducted by Hao et al. (2019) , which surveyed 74 cities across China and estimated an average VSL of 1.525 million yuan (in 2016 prices). This study was selected because it is the most recent and nationally representative VSL study available for China. The contingent valuation method used in that study asked respondents about their willingness to pay for mortality risk reduction, which is a standard and widely accepted approach for VSL estimation in environmental economics.

**Point 2: Rationale for income adjustment**

We have now explicitly stated the rationale for adjusting the baseline VSL based on local income. The adjustment accounts for the well‑established empirical finding that willingness to pay for mortality risk reduction increases with income—individuals with higher incomes are generally willing to pay more to reduce their mortality risk. This is a standard practice in the environmental economics and health economics literature when transferring VSL estimates across regions with different income levels.

The following text has been added to the Methods section:

"We adjusted the baseline VSL to each county in Chengdu using per capita annual disposable income, based on the rationale that individuals' willingness to pay for mortality risk reduction is positively correlated with their income level. This income adjustment is standard practice in VSL transfer studies and ensures that the economic burden estimates reflect the local economic context and willingness- to -pay capacity of each county's population." **(Line 171-176)**

**Point 3: Key assumptions underlying the transfer of VSL across regions**

We have now explicitly stated the key assumptions underlying our VSL transfer approach:

Income elasticity assumption: The relationship between VSL and income follows a constant elasticity function, with an income elasticity of 1. This value is derived from the same study and is consistent with the range of elasticities (0.8–1.0) commonly used in the environmental economics literature for China.

Preference homogeneity assumption: The transfer assumes that, after adjusting for income, the underlying preferences for mortality risk reduction are comparable across regions in China. This is a standard assumption in benefit transfer methods and is necessary when detailed site‑specific VSL studies are unavailable.

Price level stability assumption: The baseline VSL, reported in 2016 prices, is adjusted using real income growth, and we assume that the relative price levels between 2016 and 2022 are adequately captured by changes in per capita disposable income. This avoids double‑counting inflation effects.

Applicability to temperature‑related mortality assumption: The VSL estimates derived from general mortality risk reduction are assumed to be applicable to temperature‑related mortality. This assumes that individuals value mortality risk reductions similarly regardless of the specific cause of death—a common assumption in the literature when cause‑specific VSL estimates are unavailable.

**Point 4: Additional clarification for non‑economist readers**

To further improve interpretability for a broader readership, we have also added a brief plain‑language explanation of what VSL represents:

"The VSL does not represent the monetary value of an individual life, but rather reflects a population‑based measure of how much individuals in a given population are collectively willing to pay for small reductions in mortality risk. In this study, VSL is used as a standard metric to quantify the aggregate economic burden of premature deaths attributable to non‑optimal temperatures." **(Line 163-168)**

4. The discussion could be slightly strengthened by further emphasizing the climatic and geographic characteristics of Chengdu (e.g., basin topography, humidity, and possible heat retention effects) to better contextualize the findings.

**AUTHOR RESPONSE:** We thank the reviewer for this constructive suggestion. We fully agree that highlighting Chengdu's unique climatic and geographic characteristics will help readers better understand and contextualize our findings. In response, we have added a dedicated paragraph in the Discussion section that explicitly addresses these features and their implications for our results.

"The present findings should be interpreted in the context of Chengdu’s unique geographical and climatic characteristics. As a city situated in the topographically enclosed western Sichuan Basin and surrounded by the Qionglai and Longquan mountain ranges, Chengdu features distinct local meteorological conditions that differ from those of other Chinese cities; the surrounding mountain barriers restrict atmospheric circulation, triggering frequent autumn and winter temperature inversions and the accumulation of air pollutants and atmospheric moisture. The study-period annual average relative humidity of 79.0% reflects the basin’s humid climate, which aggravates physiological stress induced by non-optimal temperatures under both cold and hot conditions: high humidity increases air thermal conductivity to accelerate bodily heat loss and amplify cold stress in cool environments, while it inhibits sweat evaporation and cutaneous heat dissipation, elevating the risk of heat-related illnesses in hot environments. Additionally, the bowl-shaped basin terrain strengthens the urban heat island effect by limiting horizontal air movement and trapping surface heat, especially during summer nights, further exacerbating the health hazards of hot temperatures for vulnerable populations in densely urbanized areas. These unique geographical and climatic interactions jointly explain the prominent mortality burden attributable to non-optimal temperatures observed in this study, highlighting the need for targeted, context-specific public health adaptation strategies that fully account for the combined effects of temperature, humidity, and air pollution." **(Line 334-353)**

5. The use of a distributed lag non-linear model (DLNM) with quasi-Poisson regression is appropriate. However, some modeling choices would benefit from clearer explanation. In particular the rationale for selecting a maximum lag of 14 days is not clearly stated. The choice of degrees of freedom for spline functions (e.g., time trend) could be briefly justified. Also, it would be helpful to clarify whether alternative model specifications were tested.

**AUTHOR RESPONSE:** We thank the reviewer for this constructive comment. We have revised the Methods section to provide clearer explanations of our modeling choices:

First, the 14-day maximum lag was chosen based on biological plausibility—cold-induced physiological responses can take days to accumulate and culminate in acute events—and is consistent with major multi-city studies in China and the MCC Collaborative Research Network. Sensitivity analyses using 7- and 21-day lags yielded nearly identical results (Table S2), confirming robustness.

Second, the degrees of freedom for the time trend (7 df/year) and relative humidity (3 df) were selected via Akaike Information Criterion (AIC) optimization, balancing model fit and over-fitting. This follows standard practice in time-series environmental epidemiology.

Third, we tested a range of alternative specifications, including varying lag periods, and adjustment with/without air pollutants. All results remained consistent with the main findings (Table S2).

We have incorporated these clarifications into the revised Methods section and trust this addresses the reviewer's concerns. **(Line 111-117)**
