# Supplementary material for: Assessment of temperature on mortality burden and economic impacts in a basin-located mega-city
Source: PLoS One. 2026 Aug 12;21(8):e0355835. doi: 10.1371/journal.pone.0355835 (PMC13465830; doi:10.1371/journal.pone.0355835)
Supplement: Peer review history

# Peer review: Decision Letter 0

**DOI:** 10.1371/journal.pone.0355835.r001

**Authors:** Jiafu Li

29 Jun 2026

Dear Dr. Kuang,

Thank you for submitting your manuscript to PLOS ONE. After careful consideration, we feel that it has merit but does not fully meet PLOS ONE’s publication criteria as it currently stands. Therefore, we invite you to submit a revised version of the manuscript that addresses the points raised during the review process.

Please submit your revised manuscript by Aug 12 2026 11:59PM. If you will need more time than this to complete your revisions, please reply to this message or contact the journal office at plosone@plos.org. When you're ready to submit your revision, log on to https://www.editorialmanager.com/pone/ and select the 'Submissions Needing Revision' folder to locate your manuscript file.

- A letter that responds to each point raised by the academic editor and reviewer(s). You should upload this letter as a separate file labeled 'Response to Reviewers'.
- A marked-up copy of your manuscript that highlights changes made to the original version. You should upload this as a separate file labeled 'Revised Manuscript with Track Changes'.
- An unmarked version of your revised paper without tracked changes. You should upload this as a separate file labeled 'Manuscript'.

If you would like to make changes to your financial disclosure, please include your updated statement in your cover letter. Guidelines for resubmitting your figure files are available below the reviewer comments at the end of this letter.

If applicable, we recommend that you deposit your laboratory protocols in protocols.io to enhance the reproducibility of your results. Protocols.io assigns your protocol its own identifier (DOI) so that it can be cited independently in the future. For instructions see: https://journals.plos.org/plosone/s/submission-guidelines#loc-laboratory-protocols. Additionally, PLOS ONE offers an option for publishing peer-reviewed Lab Protocol articles, which describe protocols hosted on protocols.io. Read more information on sharing protocols at https://plos.org/protocols?utm_medium=editorial-email&utm_source=authorletters&utm_campaign=protocols.

As the corresponding author, your ORCID iD is verified in the submission system and will appear in the published article. PLOS supports the use of ORCID, and we encourage all coauthors to register for an ORCID iD and use it as well. Please encourage your coauthors to verify their ORCID iD within the submission system before final acceptance, as unverified ORCID iDs will not appear in the published article. *Only*  the individual author can complete the verification step; PLOS staff *cannot*  verify ORCID iDs on behalf of authors.

We look forward to receiving your revised manuscript.

Kind regards,

Jiafu Li, Ph.D

Academic Editor

PLOS One

Journal Requirements:

When submitting your revision, we need you to address these additional requirements.

1. Please ensure that your manuscript meets PLOS ONE's style requirements, including those for file naming. The PLOS ONE style templates can be found at

https://journals.plos.org/plosone/s/file?id=wjVg/PLOSOne_formatting_sample_main_body.pdf and

https://journals.plos.org/plosone/s/file?id=ba62/PLOSOne_formatting_sample_title_authors_affiliations.pdf

2. When completing the data availability statement of the submission form, you indicated that you will make your data available on acceptance. We strongly recommend all authors decide on a data sharing plan before acceptance, as the process can be lengthy and hold up publication timelines. Please note that, though access restrictions are acceptable now, your entire data will need to be made freely accessible if your manuscript is accepted for publication. This policy applies to all data except where public deposition would breach compliance with the protocol approved by your research ethics board. If you are unable to adhere to our open data policy, please kindly revise your statement to explain your reasoning and we will seek the editor's input on an exemption. Please be assured that, once you have provided your new statement, the assessment of your exemption will not hold up the peer review process.

3. Please amend your authorship list in your manuscript file to include author Dr. Wei Huang.

4. Please ensure that you refer to Figure 2 in your text as, if accepted, production will need this reference to link the reader to the figure.

5. We note that Figure 4 in your submission contain [map/satellite] images which may be copyrighted. All PLOS content is published under the Creative Commons Attribution License (CC BY 4.0), which means that the manuscript, images, and Supporting Information files will be freely available online, and any third party is permitted to access, download, copy, distribute, and use these materials in any way, even commercially, with proper attribution. For these reasons, we cannot publish previously copyrighted maps or satellite images created using proprietary data, such as Google software (Google Maps, Street View, and Earth). For more information, see our copyright guidelines: http://journals.plos.org/plosone/s/licenses-and-copyright.

We require you to either (1) present written permission from the copyright holder to publish these figures specifically under the CC BY 4.0 license, or (2) remove the figures from your submission:

1. You may seek permission from the original copyright holder of Figure 4 to publish the content specifically under the CC BY 4.0 license.

We recommend that you contact the original copyright holder with the Content Permission Form (http://journals.plos.org/plosone/s/file?id=7c09/content-permission-form.pdf) and the following text:

“I request permission for the open-access journal PLOS ONE to publish XXX under the Creative Commons Attribution License (CCAL) CC BY 4.0 (http://creativecommons.org/licenses/by/4.0/). Please be aware that this license allows unrestricted use and distribution, even commercially, by third parties. Please reply and provide explicit written permission to publish XXX under a CC BY license and complete the attached form.”

Please upload the completed Content Permission Form or other proof of granted permissions as an ""Other"" file with your submission.

In the figure caption of the copyrighted figure, please include the following text: “Reprinted from [ref] under a CC BY license, with permission from [name of publisher], original copyright [original copyright year].”

2. If you are unable to obtain permission from the original copyright holder to publish these figures under the CC BY 4.0 license or if the copyright holder’s requirements are incompatible with the CC BY 4.0 license, please either i) remove the figure or ii) supply a replacement figure that complies with the CC BY 4.0 license. Please check copyright information on all replacement figures and update the figure caption with source information. If applicable, please specify in the figure caption text when a figure is similar but not identical to the original image and is therefore for illustrative purposes only.

The following resources for replacing copyrighted map figures may be helpful:

USGS National Map Viewer (public domain): http://viewer.nationalmap.gov/viewer/

The Gateway to Astronaut Photography of Earth (public domain): http://eol.jsc.nasa.gov/sseop/clickmap/

Maps at the CIA (public domain): https://www.cia.gov/library/publications/the-world-factbook/index.html and https://www.cia.gov/library/publications/cia-maps-publications/index.html

NASA Earth Observatory (public domain): http://earthobservatory.nasa.gov/

Landsat: http://landsat.visibleearth.nasa.gov/

USGS EROS (Earth Resources Observatory and Science (EROS) Center) (public domain): http://eros.usgs.gov/#

Natural Earth (public domain): http://www.naturalearthdata.com/

6. If the reviewer comments include a recommendation to cite specific previously published works, please review and evaluate these publications to determine whether they are relevant and should be cited. There is no requirement to cite these works unless the editor has indicated otherwise.

[Note: HTML markup is below. Please do not edit.]

Reviewers' comments:

Reviewer's Responses to Questions

**Comments to the Author**

1. Is the manuscript technically sound, and do the data support the conclusions?

Reviewer #1: Partly

Reviewer #2: Yes

**********

2. Has the statistical analysis been performed appropriately and rigorously?

Reviewer #1: Yes

Reviewer #2: Yes

**********

3. Have the authors made all data underlying the findings in their manuscript fully available?

The PLOS Data policy

Reviewer #1: Yes

Reviewer #2: Yes

**********

4. Is the manuscript presented in an intelligible fashion and written in standard English?

Reviewer #1: No

Reviewer #2: Yes

**********

Reviewer #1: The manuscript addresses an important public health topic by estimating the mortality burden and economic impact of non-optimal temperatures in Chengdu.

However, several issues should be addressed before publication.

1. The novelty is somewhat limited because similar DLNM-based temperature-mortality studies have already been conducted in China and in the Sichuan Basin. The authors should clarify more explicitly what new scientific contribution this Chengdu-specific study provides beyond local estimation.

2. The 2022 excess death and VSL-based economic burden analysis needs clearer methodological explanation. The manuscript should provide the full VSL adjustment formula, all parameter values, units, income elasticity, and uncertainty propagation. It should also justify applying the 2015–2021 exposure-response relationship to the extreme temperature conditions in 2022.

3. Several reporting errors should be corrected. For example, Table 2 contains an apparent error for male extreme heat RR, reported as 1.05 (1.10–1.10). The authors should carefully check all tables, figures, and formulas.

4. The manuscript requires language editing. There are typographical and grammatical errors such as “quasi-Posson,” “form” instead of “from,” and repeated wording such as “the the.” The figures also need improvement, especially in font size and readability.

Overall, the study is potentially publishable after major revision.

Reviewer #2: General assessment

This manuscript investigates the association between ambient temperature and non-accidental mortality in Chengdu, China, using a distributed lag non-linear model (DLNM), and further estimates attributable mortality and associated economic impacts based on the value of statistical life (VSL) approach. The topic is timely and relevant in the context of climate change and public health. The dataset is large, and the analytical approach is appropriate and consistent with existing epidemiological literature. Overall, the manuscript is well structured and presents interesting findings regarding temperature-related mortality burden and economic loss.

However, several aspects of the manuscript would benefit from clarification to improve transparency, readability, and reproducibility. These issues are mainly related to methodological description, interpretation, and presentation rather than fundamental flaws in the study design. Therefore, I recommend minor revision.

Major comments

1. The manuscript mentions sensitivity analyses (e.g., alternative lag structures and adjustment for air pollutants), yet the presentation of these results is limited. I suggest adding a concise summary—either in the Results section or in supplementary materials—indicating whether the main findings remained robust across these variations.

2. Interpretation of attributable estimates The manuscript reports attributable fractions and excess deaths associated with non-optimal temperatures. While these estimates are useful, it may help readers if the authors briefly clarify that these are model-based estimates derived from statistical associations under specific assumptions.

3. The value of statistical life (VSL) approach is appropriate for estimating economic burden, but some methodological details could be clarified for broader readership. It would be helpful to briefly explain the source of the VSL estimate, the rationale for income adjustment and key assumptions underlying the transfer of VSL across regions. This would improve interpretability for non-economists.

4. The discussion could be slightly strengthened by further emphasizing the climatic and geographic characteristics of Chengdu (e.g., basin topography, humidity, and possible heat retention effects) to better contextualize the findings.

5. The use of a distributed lag non-linear model (DLNM) with quasi-Poisson regression is appropriate. However, some modeling choices would benefit from clearer explanation. In particular the rationale for selecting a maximum lag of 14 days is not clearly stated. The choice of degrees of freedom for spline functions (e.g., time trend) could be briefly justified. Also, it would be helpful to clarify whether alternative model specifications were tested.

Recommendation

Minor revision

The manuscript addresses an important public health topic and uses appropriate methodology. The required revisions are mainly related to clarification of methods, improved explanation of assumptions. Addressing these points will improve the clarity and accessibility of the manuscript.

**********

what does this mean?). If published, this will include your full peer review and any attached files.

If you choose “no”, your identity will remain anonymous but your review may still be made public.

**Do you want your identity to be public for this peer review?**  For information about this choice, including consent withdrawal, please see our Privacy Policy

Reviewer #1: No

Reviewer #2: No

**********

[NOTE: If reviewer comments were submitted as an attachment file, they will be attached to this email and accessible via the submission site. Please log into your account, locate the manuscript record, and check for the action link "View Attachments". If this link does not appear, there are no attachment files.]

To ensure your figures meet our technical requirements, please review our figure guidelines: https://journals.plos.org/plosone/s/figures

You may also use PLOS’s free figure tool, NAAS, to help you prepare publication quality figures: https://journals.plos.org/plosone/s/figures#loc-tools-for-figure-preparation.

NAAS will assess whether your figures meet our technical requirements by comparing each figure against our figure specifications.

# Author comment: Author response to Decision Letter 1

**DOI:** 10.1371/journal.pone.0355835.r002

9 Jul 2026

Dear Reviewers,

We greatly appreciate your comments and suggestions. In response to the reviewers' feedback, we have addressed each point raised by the academic editor and the reviewers. Additionally, we have made some changes to the manuscript, which are highlighted for your convenience.

Authors Responses to Journal Requirements

When submitting your revision, we need you to address these additional requirements.

1. Please ensure that your manuscript meets PLOS ONE's style requirements, including those for file naming. The PLOS ONE style templates can be found at

https://journals.plos.org/plosone/s/file?id=wjVg/PLOSOne_formatting_sample_main_body.pdf and

https://journals.plos.org/plosone/s/file?id=ba62/PLOSOne_formatting_sample_title_authors_affiliations.pdf

AUTHOR RESPONSE: We have thoroughly reviewed the PLOS ONE style templates and confirm that the manuscript now fully complies with all formatting guidelines, including file naming conventions. All sections (title page, main text, references, and figures) have been adjusted to meet the journal's requirements.

2. When completing the data availability statement of the submission form, you indicated that you will make your data available on acceptance. We strongly recommend all authors decide on a data sharing plan before acceptance, as the process can be lengthy and hold up publication timelines. Please note that, though access restrictions are acceptable now, your entire data will need to be made freely accessible if your manuscript is accepted for publication. This policy applies to all data except where public deposition would breach compliance with the protocol approved by your research ethics board. If you are unable to adhere to our open data policy, please kindly revise your statement to explain your reasoning and we will seek the editor's input on an exemption. Please be assured that, once you have provided your new statement, the assessment of your exemption will not hold up the peer review process.

AUTHOR RESPONSE: We confirm our commitment to PLOS ONE's open data policy. Our complete dataset will be deposited in the Supporting information (S1 Table. The raw data needed to replicate the findings of this study) of this article upon acceptance.

3. Please amend your authorship list in your manuscript file to include author Dr. Wei Huang.

AUTHOR RESPONSE: We sincerely thank the editor for this reminder. We have now added Dr. Wei Huang to the authorship list in the revised manuscript file, and all coauthors have confirmed their agreement with this change. The updated author list appears on the title page, and the corresponding author information has been checked accordingly.

4. Please ensure that you refer to Figure 2 in your text as, if accepted, production will need this reference to link the reader to the figure.

AUTHOR RESPONSE: We confirm that Figure 2 is now correctly cited in the text, first appearing in the Results section (specifically in the “Associations between temperature and mortality” subsection). (Line 212)

5.We note that Figure 4 in your submission contain [map/satellite] images which may be copyrighted. All PLOS content is published under the Creative Commons Attribution License (CC BY 4.0), which means that the manuscript, images, and Supporting Information files will be freely available online, and any third party is permitted to access, download, copy, distribute, and use these materials in any way, even commercially, with proper attribution. For these reasons, we cannot publish previously copyrighted maps or satellite images created using proprietary data, such as Google software (Google Maps, Street View, and Earth). For more information, see our copyright guidelines: http://journals.plos.org/plosone/s/licenses-and-copyright.

AUTHOR RESPONSE: We thank the editor for bringing this copyright issue to our attention. In compliance with PLOS’s copyright and licensing policy, we have removed the map images that were originally shown in Figure 4A. The revised Figure 4 now includes only the economic burden data (previously Figure 4B), presented as a bar chart with error bars and the VSL‑to‑GDP ratio line. We have also updated the figure caption and the corresponding text in the Results and Discussion sections to accurately describe the revised figure.

6.If the reviewer comments include a recommendation to cite specific previously published works, please review and evaluate these publications to determine whether they are relevant and should be cited. There is no requirement to cite these works unless the editor has indicated otherwise.

AUTHOR RESPONSE: We appreciate the editor’s reminder. After careful examination of all reviewer comments, we found that no specific prior publications were recommended for citation. Accordingly, we have not made any changes to the reference list in response to this point.

Author’s Responses to Review Comments

Comments from Reviewer #1:

1. The novelty is somewhat limited because similar DLNM-based temperature-mortality studies have already been conducted in China and in the Sichuan Basin. The authors should clarify more explicitly what new scientific contribution this Chengdu-specific study provides beyond local estimation.

AUTHOR RESPONSE: We thank the reviewer for this constructive comment. We agree that similar DLNM-based studies on temperature and mortality have been conducted in China and the Sichuan Basin. However, we would like to clarify the specific and novel contributions of our study, which extend beyond a mere local estimation:

First, this is the first study to systematically quantify the economic burden of mortality attributable to non-optimal temperatures in Chengdu. While previous studies have examined the mortality burden of temperature in Chengdu, none have comprehensively assessed the associated economic losses using the value of statistical life (VSL) approach at the county level. Our study fills this critical gap by estimating that 11,316 excess deaths in 2022 resulted in an economic loss of 21.428 billion RMB , providing essential evidence for cost‑effectiveness analyses of public health interventions and climate adaptation policies. To our knowledge, this economic dimension has not been previously addressed for Chengdu.

Second, our county‑level spatial analysis reveals significant intra‑urban heterogeneity in both mortality burden and economic impact across Chengdu's 23 counties, with the ratio of VSL to GDP ranging from 3.65‰ in Wuhou to 26.40‰ in Dongbuxinqu. This granular spatial evidence can guide targeted, locally‑tailored adaptation strategies—a level of policy‑relevant detail not available from broader regional or national studies.

Third, our study updates and expands the evidence base with a more recent and longer time series (2015-2021). Given the accelerating pace of climate change—with 2022 being one of the hottest years on record in China—and ongoing urbanization intensifying the urban heat island effect, updated evidence is urgently needed. Our findings reveal a notable shift: the cold‑attributable fraction decreased from 9.96% (2011-2014) to 5.82% (2015-2021), while the heat‑attributable fraction increased from 0.97% to 1.42%. This temporal trend, which we explicitly discuss in the manuscript, carries important implications for climate adaptation planning and demonstrates the value of updated local evidence.

We have revised the Discussion sections to more explicitly articulate these contributions, and we hope the reviewer finds our clarifications convincing:

“This study offers several notable innovations to the existing body of evidence on temperature-related mortality. First, to the best of our knowledge, this is the first study to systematically quantify the economic burden of mortality attributable to non-optimal temperatures in Chengdu using the VSL framework. This economic perspective has long been absent from local temperature health research, yet it is essential for supporting cost-effectiveness assessments of public health interventions and optimizing climate adaptation policy formulation. Second, our fine-scale county-level spatial analysis identified substantial intra-urban heterogeneity in temperature-attributable mortality burdens and corresponding economic impacts across Chengdu’s 23 administrative counties. Such refined spatial evidence provides a solid foundation for developing differentiated, locality-targeted climate adaptation strategies. Third, this study updates and enriches the temporal evidence base by adopting a relatively recent and prolonged observational period (2015-2021), which is particularly valuable amid ongoing climate change acceleration and rapid urbanization processes. Collectively, these advances extend beyond localized epidemiological estimation and generate refined, policy-relevant evidence for climate risk adaptation in densely urbanized mega-cities” (Line 485-501)

2. The 2022 excess death and VSL-based economic burden analysis needs clearer methodological explanation. The manuscript should provide the full VSL adjustment formula, all parameter values, units, income elasticity, and uncertainty propagation. It should also justify applying the 2015–2021 exposure-response relationship to the extreme temperature conditions in 2022.

AUTHOR RESPONSE: We thank the reviewer for this valuable comment. We agree that the methodological details for the 2022 excess death and economic burden estimation require clearer exposition. We have now substantially revised the Methods section to address each of the points raised, and we have also added relevant discussions in the Results and Discussion sections. Below we respond point‑by‑point:

Point 1: Full VSL adjustment formula, parameter values, units, and income elasticity

We have now provided the complete VSL adjustment formula with all parameter values and units explicitly stated. The formula is as follows:

VSLi=EDi×e (3)

Where, VSLi is the estimated VSL attributable to the 2022 non-optimal temperature for city i (100 million yuan); EDi is the estimated number of excess non-accidental deaths during 2022 in county i; VSL is 1.525 million yuan adopted from the Chinese study. INCOME2022i is the per capita annual income for county i in 2022 (ten thousand yuan); INCOME2016 is 4.35 ten thousand yuan adopted from the Chinese study as the national average per capita annual income in 2016. e is 1 as the income elasticity.

The baseline VSL (152.5 ten thousand yuan) was adopted from the study by Hao et al. (2019), which estimated VSL for 74 Chinese cities using a contingent valuation method. The income elasticity of 1 is derived from the same study, consistent with the commonly used value in the environmental economics literature for China. We have added this full specification to the Materials and Methods section (subsection: "Excess non-accidental deaths and economic impact attributable to 2022 non-optimal temperatures") and included a supplementary table (Table S3) summarizing all parameters.

Table S3.Excess death and economic impact of 2022

County Population

(10,000) VSL

(10,000 yuan) Income-2016

(10,000 yuan) Income-2022

(10,000 yuan) GDP

(100 million yuan) Excess death VSL-2022

(100 million yuan) VSL-2022/GDP

(‰)

Jinjiang 91.44 152.5 4.35 5.7629 1304.1885 486.57 (285.02-675.49 ) 9.83 (5.76-13.65 ) 7.54 (4.42-10.46 )

Qinyang 96.73 152.5 4.35 5.7479 1496.3926 514.72 (301.51-714.57 ) 10.37 (6.08-14.4 ) 6.93 (4.06-9.62 )

Jinniu 128.36 152.5 4.35 5.6108 1499.1307 683.03 (400.1-948.23 ) 13.44 (7.87-18.65 ) 8.96 (5.25-12.44 )

Wuhou 121.98 152.5 4.35 5.8172 3629.2805 649.08 (380.21-901.1 ) 13.24 (7.75-18.38 ) 3.65 (2.14-5.06 )

Chenghua 140.29 152.5 4.35 5.7563 1360.5811 746.51 (437.29-1036.36 ) 15.06 (8.82-20.91 ) 11.07 (6.49-15.37 )

Longquanyi 136.37 152.5 4.35 5.5072 1545.7433 725.66 (425.07-1007.4 ) 14.01 (8.21-19.45 ) 9.06 (5.31-12.58 )

Qingbaijiang 50.6 152.5 4.35 5.2344 650.6909 269.25 (157.72-373.79 ) 4.94 (2.89-6.86 ) 7.59 (4.45-10.54 )

Xindu 157.73 152.5 4.35 5.6632 1032.5907 839.32 (491.65-1165.19 ) 16.66 (9.76-23.13 ) 16.14 (9.45-22.4 )

Wenjiang 99.87 152.5 4.35 5.6066 717.1079 531.43 (311.3-737.76 ) 10.45 (6.12-14.5 ) 14.57 (8.53-20.22 )

Shuangliu 150.1 152.5 4.35 5.8113 1911.0968 798.72 (467.87-1108.83 ) 16.27 (9.53-22.59 ) 8.51 (4.99-11.82 )

Pidu 139.47 152.5 4.35 5.5922 1399.2049 742.15 (434.73-1030.3 ) 14.55 (8.52-20.2 ) 10.4 (6.09-14.44 )

Xinjin 37.37 152.5 4.35 4.9696 469.1486 198.85 (116.48-276.06 ) 3.46 (2.03-4.81 ) 7.38 (4.33-10.25 )

Jianyang 73.6 152.5 4.35 4.5281 672.9895 391.64 (229.41-543.7 ) 6.22 (3.64-8.63 ) 9.24 (5.41-12.82 )

Dujiangyan 71.46 152.5 4.35 4.5502 483.6648 380.25 (222.74-527.89 ) 6.07 (3.55-8.42 ) 12.54 (7.35-17.41 )

Pengzhou 78.01 152.5 4.35 4.5547 638.9173 415.11 (243.16-576.28 ) 6.63 (3.88-9.2 ) 10.37 (6.08-14.4 )

Qionglai 60.13 152.5 4.35 4.5844 400.6312 319.97 (187.43-444.2 ) 5.14 (3.01-7.14 ) 12.84 (7.52-17.82 )

Chongzhou 74.18 152.5 4.35 4.711 461.7951 394.73 (231.22-547.99 ) 6.52 (3.82-9.05 ) 14.12 (8.27-19.6 )

Jintang 80.31 152.5 4.35 4.7341 602.9301 427.35 (250.33-593.27 ) 7.09 (4.15-9.85 ) 11.76 (6.89-16.33 )

Dayi 51.02 152.5 4.35 4.5296 329.88 271.49 (159.03-376.9 ) 4.31 (2.53-5.99 ) 13.07 (7.66-18.14 )

Pujiang 25.74 152.5 4.35 4.4318 211.5396 136.97 (80.23-190.15 ) 2.13 (1.25-2.95 ) 10.06 (5.89-13.97 )

Tianfuxinqu 91.26 152.5 4.35 5.6686 669 485.62 (284.46-674.16 ) 9.65 (5.65-13.4 ) 14.43 (8.45-20.03 )

Dongbuxinqu 40.1 152.5 4.35 4.4594 126.3 213.38 (124.99-296.23 ) 3.34 (1.95-4.63 ) 26.41 (15.47-36.67 )

Gaoxin 130.39 152.5 4.35 6.1277 3015.8 693.83 (406.43-963.22 ) 14.9 (8.73-20.69 ) 4.94 (2.9-6.86 )

Point 2: Uncertainty propagation

We have now explicitly described the uncertainty propagation method. The 95% confidence intervals for excess deaths and VSL estimates were derived through the following procedure:

We generated 1,000 random draws from the multivariate normal distribution of the DLNM coefficient estimates, using the variance‑covariance matrix of the model coefficients.

For each draw, we calculated the RR for each day of 2022 and estimated the corresponding excess deaths using Equation (2).

We then computed the VSL for each draw using Equation (3) with the county‑specific income data.

The 2.5th and 97.5th percentiles of the 1,000 estimates were used as the 95% confidence intervals for both excess deaths and VSL.

Point 3: Justification for applying the 2015–2021 exposure‑response relationship to 2022 extreme temperature conditions

First, the 2015–2021 period was chosen specifically because it represents the most recent multi‑year baseline available prior to 2022, capturing the prevailing climate, demographic, and socioeconomic conditions most relevant to the 2022 population. Using a baseline period that immediately precedes the target year minimizes temporal confounding and ensures that the estimated exposure‑response relationship reflects the contemporary vulnerability profile of the Chengdu population.

Second, the exposure‑response function is estimated across the full temperature distribution of the baseline period (‑1.6°C to 30.5°C), which already covers the most temperature range observed in 2022 (2.2°C to 32.2°C). The 2022 extreme heat and cold conditions (30.4℃ and 5.4℃) fall within the range of temperatures experienced during 2015–2021, and our model was estimated with spline terms that allow for flexible extrapolation within this range. Thus, we are not extrapolating beyond the temperature range used for model fitting.

Third, the assumption of a stable exposure‑response relationship over short time horizons is standard practice in the environmental epidemiology literature. While we acknowledge that population vulnerability may evolve over time due to adaptation (e.g., increased air conditioning coverage, improved healthcare), such changes typically occur gradually. The two‑year gap between the end of our baseline period (2021) and the target year (2022) is sufficiently short that this assumption is reasonable.

3. Several reporting errors should be corrected. For example, Table 2 contains an apparent error for male extreme heat RR, reported as 1.05 (1.10–1.10). The authors should carefully check all tables, figures, and formulas

AUTHOR RESPONSE: We sincerely thank the reviewer

Attachment Submitted filename: Response to Reviewers.docx

# Peer review: Decision Letter 1

**DOI:** 10.1371/journal.pone.0355835.r003

**Authors:** Jiafu Li

27 Jul 2026

Assessment of temperature on mortality burden and economic impacts in a basin-located mega-city

PONE-D-26-01249R1

Dear Dr. Kuang,

We’re pleased to inform you that your manuscript has been judged scientifically suitable for publication and will be formally accepted for publication once it meets all outstanding technical requirements.

Within one week, you’ll receive an e-mail detailing the required amendments. When these have been addressed, you’ll receive a formal acceptance letter and your manuscript will be scheduled for publication.

An invoice will be generated when your article is formally accepted. Please note, if your institution has a publishing partnership with PLOS and your article meets the relevant criteria, all or part of your publication costs will be covered. Please make sure your user information is up-to-date by logging into Editorial Manager at Editorial Manager® and clicking the ‘Update My Information' link at the top of the page. For questions related to billing, please contact billing support.

If your institution or institutions have a press office, please notify them about your upcoming paper to help maximize its impact. If they’ll be preparing press materials, please inform our press team as soon as possible -- no later than 48 hours after receiving the formal acceptance. Your manuscript will remain under strict press embargo until 2 pm Eastern Time on the date of publication. For more information, please contact onepress@plos.org.

Kind regards,

Jiafu Li, Ph.D

Academic Editor

PLOS One

Additional Editor Comments (optional):

Reviewers' comments:

Reviewer's Responses to Questions

**Comments to the Author**

Reviewer #1: All comments have been addressed

**********

2. Is the manuscript technically sound, and do the data support the conclusions?

Reviewer #1: Yes

**********

3. Has the statistical analysis been performed appropriately and rigorously?

Reviewer #1: Yes

**********

4. Have the authors made all data underlying the findings in their manuscript fully available?

The PLOS Data policy

Reviewer #1: Yes

**********

5. Is the manuscript presented in an intelligible fashion and written in standard English?

Reviewer #1: Yes

**********

Reviewer #1: The research is well-structured, addresses a clear gap in current literature, and provides robust experimental evidence to support its claims. Overall, the paper is well-written, logically organized, and makes a meaningful contribution to the field.

**********

what does this mean?). If published, this will include your full peer review and any attached files.

If you choose “no”, your identity will remain anonymous but your review may still be made public.

**Do you want your identity to be public for this peer review?**  For information about this choice, including consent withdrawal, please see our Privacy Policy

Reviewer #1: No

**********

# Editor report: Acceptance letter

**DOI:** 10.1371/journal.pone.0355835.r004

**Authors:** Jiafu Li

PONE-D-26-01249R1

PLOS One

Dear Dr. Kuang,

I'm pleased to inform you that your manuscript has been deemed suitable for publication in PLOS One. Congratulations! Your manuscript is now being handed over to our production team.

At this stage, our production department will prepare your paper for publication. This includes ensuring the following:

* All references, tables, and figures are properly cited

* All relevant supporting information is included in the manuscript submission,

* There are no issues that prevent the paper from being properly typeset

You will receive further instructions from the production team, including instructions on how to review your proof when it is ready. Please keep in mind that we are working through a large volume of accepted articles, so please give us a few days to review your paper and let you know the next and final steps.

Lastly, if your institution or institutions have a press office, please let them know about your upcoming paper now to help maximize its impact. If they'll be preparing press materials, please inform our press team within the next 48 hours. Your manuscript will remain under strict press embargo until 2 pm Eastern Time on the date of publication. For more information, please contact onepress@plos.org.

You will receive an invoice from PLOS for your publication fee after your manuscript has reached the completed accept phase. If you receive an email requesting payment before acceptance or for any other service, this may be a phishing scheme. Learn how to identify phishing emails and protect your accounts at https://explore.plos.org/phishing.

If we can help with anything else, please email us at customercare@plos.org.

Thank you for submitting your work to PLOS ONE and supporting open access.

Kind regards,

PLOS ONE Editorial Office Staff

on behalf of

Dr. Jiafu Li

Academic Editor

PLOS One
